# Supplementary material for: The efficient induction of human retinal ganglion-like cells provides a platform for studying optic neuropathies
Source: Cell Mol Life Sci. 2023 Aug 4;80(8):239. doi: 10.1007/s00018-023-04890-w (PMC10403410; doi:10.1007/s00018-023-04890-w)
Supplement: Supplementary file 1 — Supplementary material (PDF 7598 kb) [file 18_2023_4890_MOESM1_ESM.pdf]

Supplementary Materials

Suppl Fig. 1

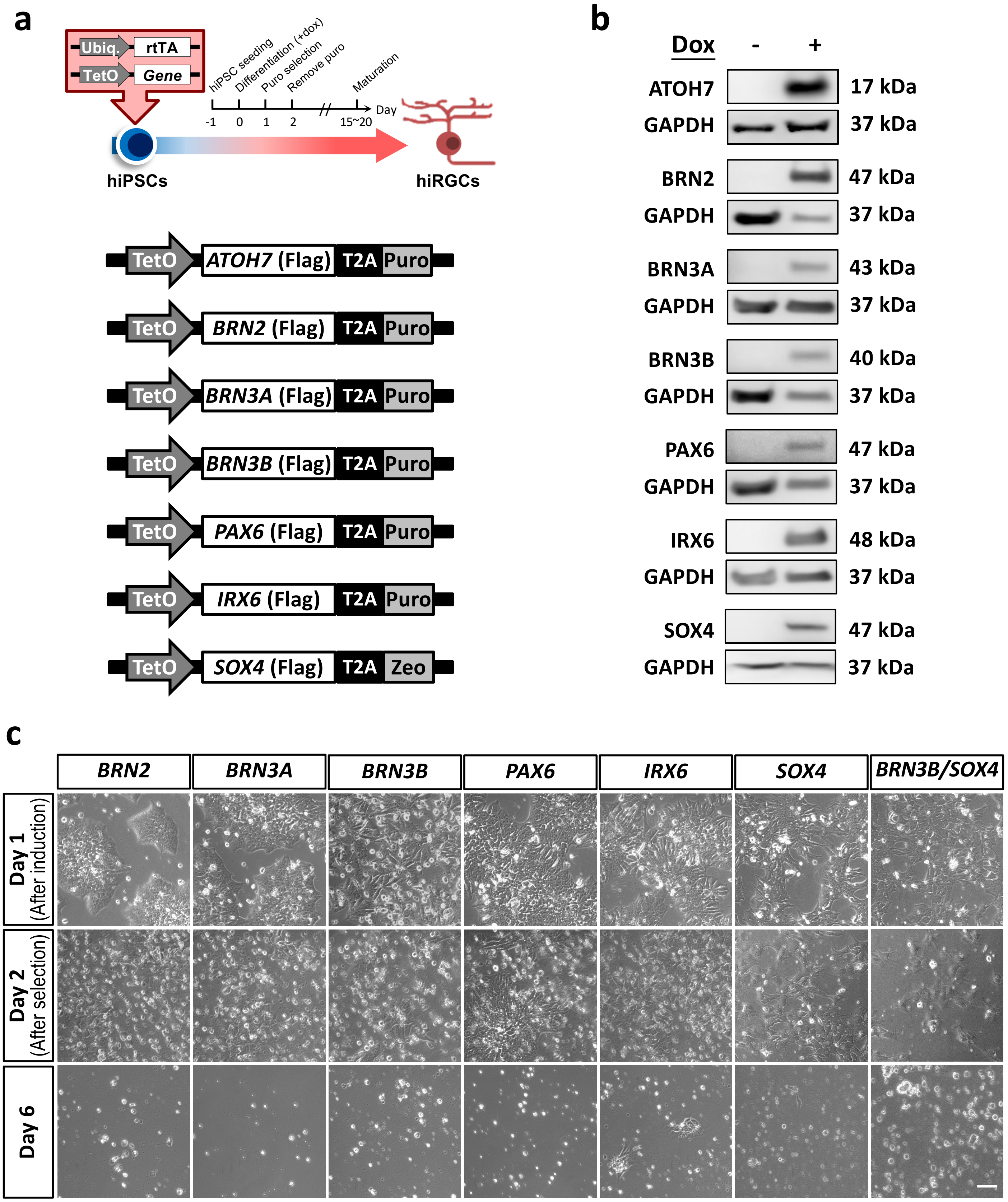
a
Ubiq. rtTA
TetO Gene
hiPSC seeding
Differentiation (+dox)
Puro selection
Remove puro
Maturation
Day
-1 0 1 2 15~20
hiPSCs
hiRGCs
TetO ATOH7 (Flag) T2A Puro
TetO BRN2 (Flag) T2A Puro
TetO BRN3A (Flag) T2A Puro
TetO BRN3B (Flag) T2A Puro
TetO PAX6 (Flag) T2A Puro
TetO IRX6 (Flag) T2A Puro
TetO SOX4 (Flag) T2A Zeo
b
Dox - +
ATOH7 17 kDa
GAPDH 37 kDa
BRN2 47 kDa
GAPDH 37 kDa
BRN3A 43 kDa
GAPDH 37 kDa
BRN3B 40 kDa
GAPDH 37 kDa
PAX6 47 kDa
GAPDH 37 kDa
IRX6 48 kDa
GAPDH 37 kDa
SOX4 47 kDa
GAPDH 37 kDa
c
BRN2
BRN3A
BRN3B
PAX6
IRX6
SOX4
BRN3B/SOX4
Day 1 (After induction)
Day 2 (After selection)
Day 6

Fig S1. Candidate factors for RGC induction from hiPSCs. (**a**) Schematic representation of the strategy used to test the candidate transcription factors (TFs) for induction of RGC differentiation from hiPSCs (upper panel). Seven TFs were selected as the candidates based on their functional roles in developmental retinogenesis and the maintenance of RGCs in vivo (lower panel). (**b**) Overexpression of the genes of interest was confirmed measuring protein levels using Western blot analysis after doxycycline induction. (**c**) Representative phase contrast images that display the morphological changes in the hiPSCs after induction with the indicated genes on days 1, 3 and 6. Most cells failed to survive under neuronal culture conditions after days 6. Scale bar, 20 µm.

Suppl Fig. 2

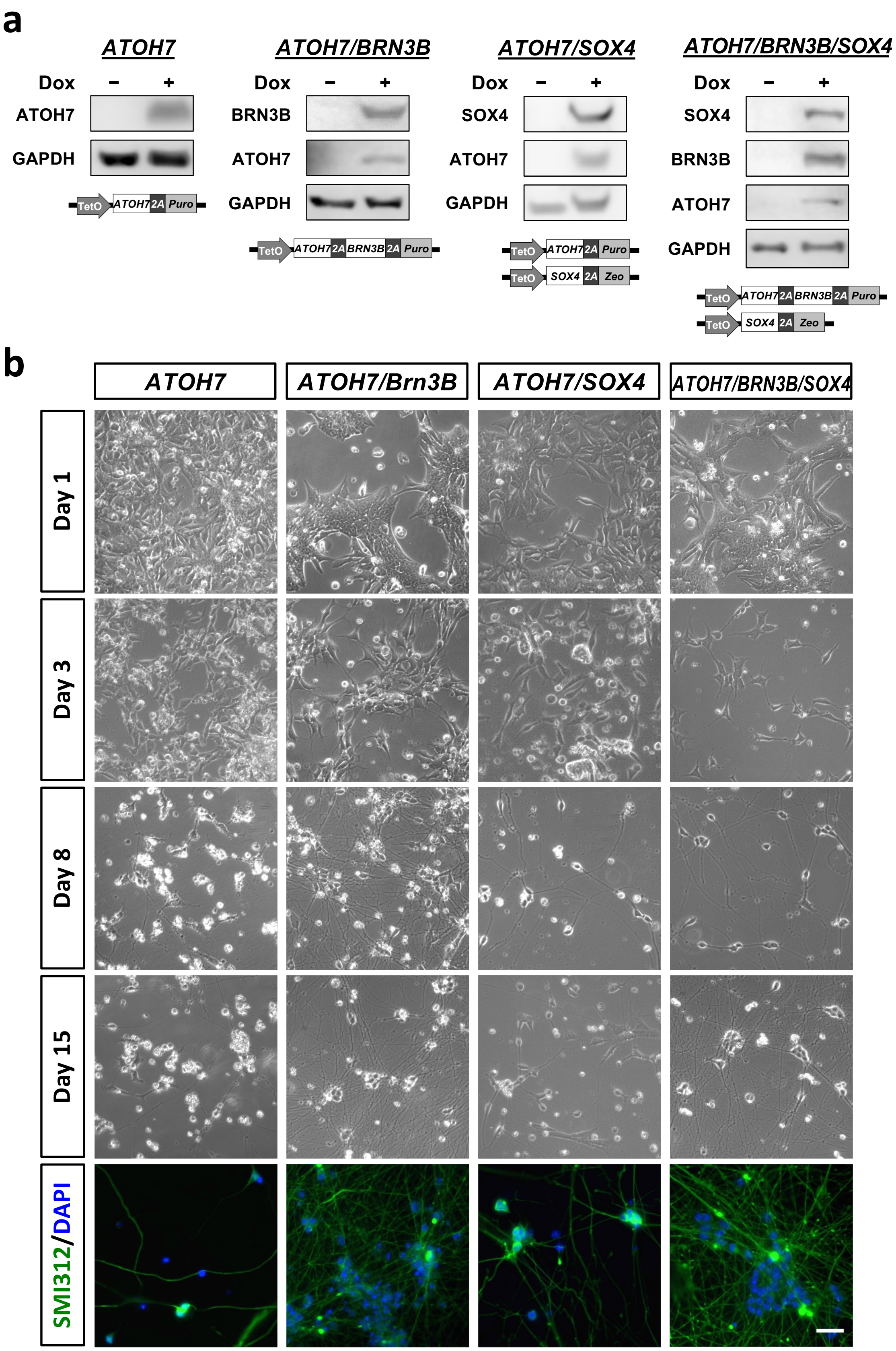

a
ATOH7
Dox – +
ATOH7
GAPDH
TetO ATOH7 2A Puro
ATOH7/BRN3B
Dox – +
BRN3B
ATOH7
GAPDH
TetO ATOH7 2A BRN3B 2A Puro
ATOH7/SOX4
Dox – +
SOX4
ATOH7
GAPDH
TetO ATOH7 2A Puro
TetO SOX4 2A Zeo
ATOH7/BRN3B/SOX4
Dox – +
SOX4
BRN3B
ATOH7
GAPDH
TetO ATOH7 2A BRN3B 2A Puro
TetO SOX4 2A Zeo
b
ATOH7
ATOH7/Brn3B
ATOH7/SOX4
ATOH7/BRN3B/SOX4
Day 1
Day 3
Day 8
Day 15
SMI312/DAPI

Fig S2. Ectopic expression of candidate TFs in hiPSCs induces neuronal-like morphology. (**a**) Overexpression of the genes of interest was confirmed by measuring protein levels using Western blot analysis after doxycycline induction. (**b**) Ectopic expression of TFs in hiPSCs induces neuronal-like morphology. Representative phase contrast images that display the morphological changes in the hiPSCs after induction with the indicated genes on days 1, 3, 8 and 15. The cells, on 15 days after doxycycline induction, displayed distinct neurite-like structures and were immunostained with the neuronal marker SMI312. Scale bar, 20 µm.

Suppl Fig. 3

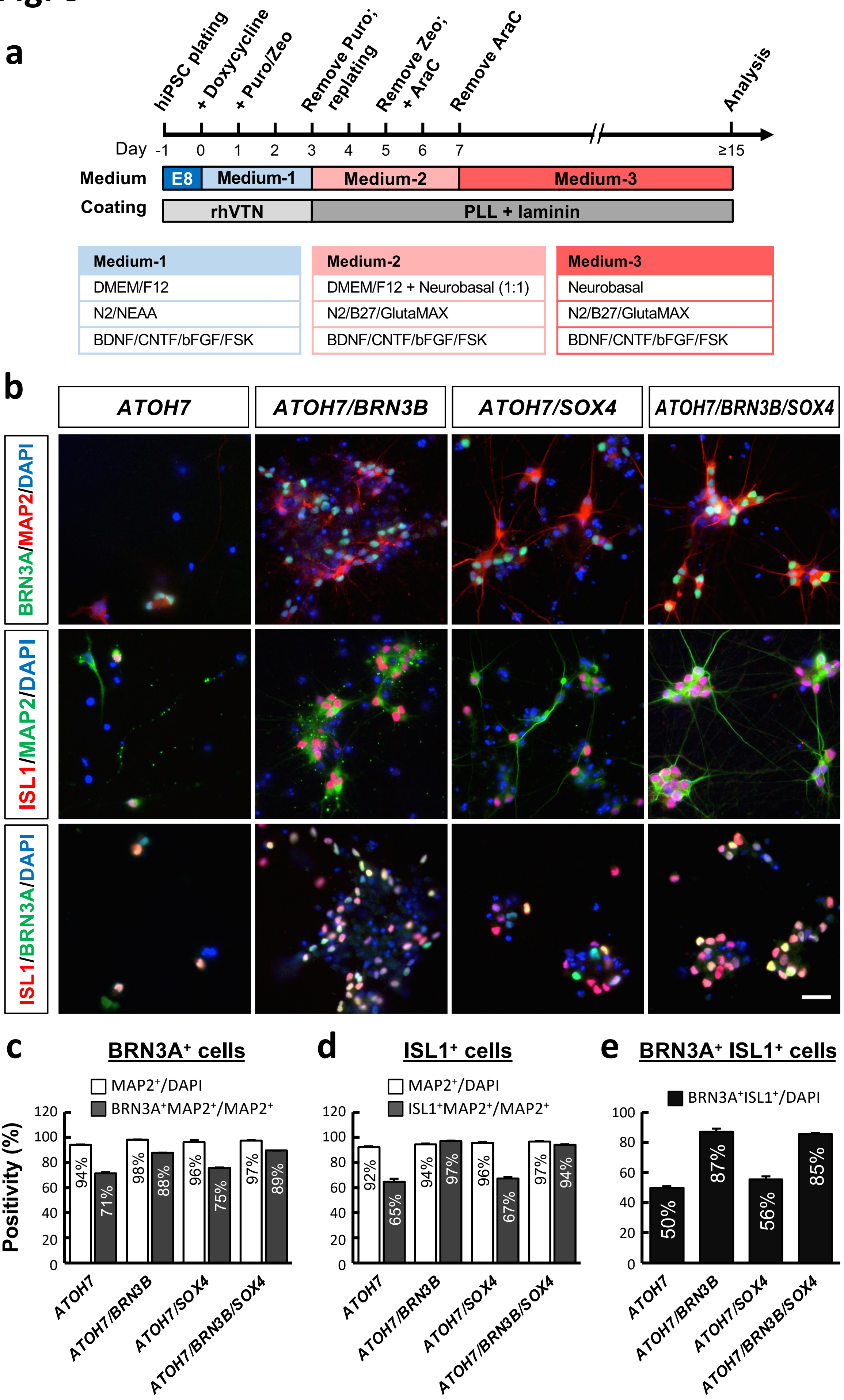
a
hiPSC plating
+ Doxycycline
+ Puro/Zeo
Remove Puro; replating
Remove Zeo; + AraC
Remove AraC
Analysis
Day -1 0 1 2 3 4 5 6 7 ≥15
Medium
E8
Medium-1
Medium-2
Medium-3
Coating
rhVTN
PLL + laminin
Medium-1
DMEM/F12
N2/NEAA
BDNF/CNTF/bFGF/FSK
Medium-2
DMEM/F12 + Neurobasal (1:1)
N2/B27/GlutaMAX
BDNF/CNTF/bFGF/FSK
Medium-3
Neurobasal
N2/B27/GlutaMAX
BDNF/CNTF/bFGF/FSK
b
ATOH7
ATOH7/BRN3B
ATOH7/SOX4
ATOH7/BRN3B/SOX4
BRN3A/MAP2/DAPI
ISL1/MAP2/DAPI
ISL1/BRN3A/DAPI
c
BRN3A⁺ cells
MAP2⁺/DAPI
BRN3A⁺MAP2⁺/MAP2⁺
Positivity (%)
0 20 40 60 80 100 120
94% 71% 98% 88% 96% 75% 97% 89%
ATOH7
ATOH7/BRN3B
ATOH7/SOX4
ATOH7/BRN3B/SOX4
d
ISL1⁺ cells
MAP2⁺/DAPI
ISL1⁺MAP2⁺/MAP2⁺
0 20 40 60 80 100 120
92% 65% 94% 97% 96% 67% 97% 94%
ATOH7
ATOH7/BRN3B
ATOH7/SOX4
ATOH7/BRN3B/SOX4
e
BRN3A⁺ ISL1⁺ cells
BRN3A⁺ISL1⁺/DAPI
0 20 40 60 80 100
50% 87% 56% 85%
ATOH7
ATOH7/BRN3B
ATOH7/SOX4
ATOH7/BRN3B/SOX4

Fig S3. Differentiation of hiPSC-derived RGC-like cells. (**a**) Flow diagram of the generation of induced RGC (iRGC). (**b**) Representative images of iRGC cells immunostained for neuronal (MAP2) and RGC (ISL1, BRN3A) markers. Scale bar, 20 µm. (**c**) Quantification of the BRN3A+ cells present among hiPSCs ectopically expressing the candidate genes. Data are presented as means ± SEM (n = 5 batches of independent differentiation). (**d**) Quantification of the ISL1+ cells present among hiPSCs ectopically expressing the candidate genes. Data are presented as means ± SEM (n = 5 batches of independent differentiation). (**e**) Quantification of the BRN3A+ISL1+ cells present among hiPSCs ectopically expressing the candidate genes. Data are presented as means ± SEM (n = 5 batches of independent differentiation).

Suppl Fig. 4

a

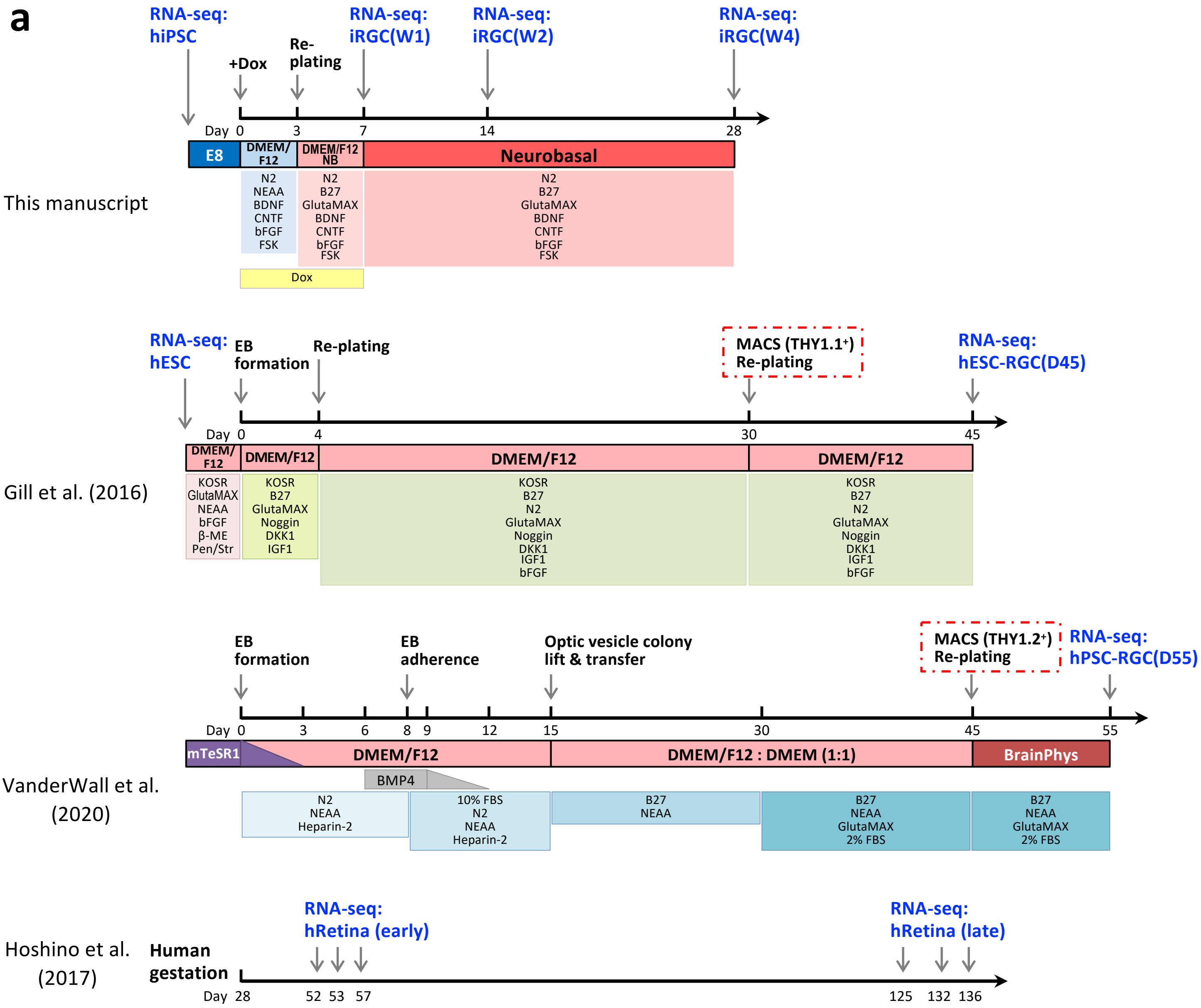

b

	This manuscript				Gill et al. (2016)		VanderWall et al. (2020)	Hoshino et al. (2017)	
**Sample**	hiPSC	iRGC(W1)	iRGC(W2)	iRGC(W4)	hESC	hESC-RGC(D45)	hiPSC-RGC(D55)	hRetina (early)	hRetina (late)
**Differ. strategy**	Direct conversion with ATOH7, BRN3B and SOX4				EB-based differentiation and THY1[+] sorting		EB-based differentiation and THY1[+] sorting	N.A.	
**Bioinform. method**	Bulk RNA-Seq (poly(A)-enriched)				Bulk RNA-Seq (poly(A)-enriched)		Bulk RNA-Seq (poly(A)-enriched)	Bulk RNA-Seq (poly(A)-enriched)	
**Platform**	Illumina NovaSeq 6000				Illumina HiSeq 2000		Illumina HiSeq 2000	Illumina HiSeq 2500	
**Layout**	Paired-end 150bp*2				Paired-end 100bp*2		Paired-end 100bp*2	Paired-end 125bp*2	
**Dataset**	GSM7517057 GSM7517058 GSM7517059 GSM7517060 GSM7517045 GSM7517046	GSM7517049 GSM7517054 GSM7517042	GSM7517047 GSM7517050 GSM7517052 GSM7517055 GSM7517040 GSM7517043	GSM7517048 GSM7517051 GSM7517053 GSM7517056 GSM7517041 GSM7517044	GSM2243611 GSM2243613 GSM2243615	GSM2243617 GSM2243619 GSM2243621	GSM4305719 GSM4305720 GSM4305721 GSM4305722	GSM2808439 GSM2808440 GSM2808441	GSM2808449 GSM2808450 GSM2808451

Fig S4. Comparison of different protocols for generation of hPSC-derived RGCs and human fetal retina. (**a**) A comparison of different protocols used for generating hPSC-derived RGC-like cells is presented. In our protocol, stable hiPSC lines carrying both pTetO-ATOH7-T2A-BRN3B-T2A-Puro and pTetO-SOX4-Zeo were induced for RGC-like cell differentiation upon treatment with doxycycline. Total RNA was extracted from hiPSC-derived RGCs at indicated time points and subjected to total RNA sequencing (RNA-seq). In the protocols of Gill et al. (2016) and VanderWall et al. (2020), hPSCs or hESCs were first differentiated into neuralized embryonic bodies (EB), followed by a switch to RGC maturation media containing N2 and B27 supplement. After sorting via magnetic anti-THY1 microbeads, these cells were kept in culture for 10 or 15 days before undergoing RNA-seq analysis. (**b**) The 9 datasets used for bioinformatic analysis are described.

Suppl Fig. 5

a

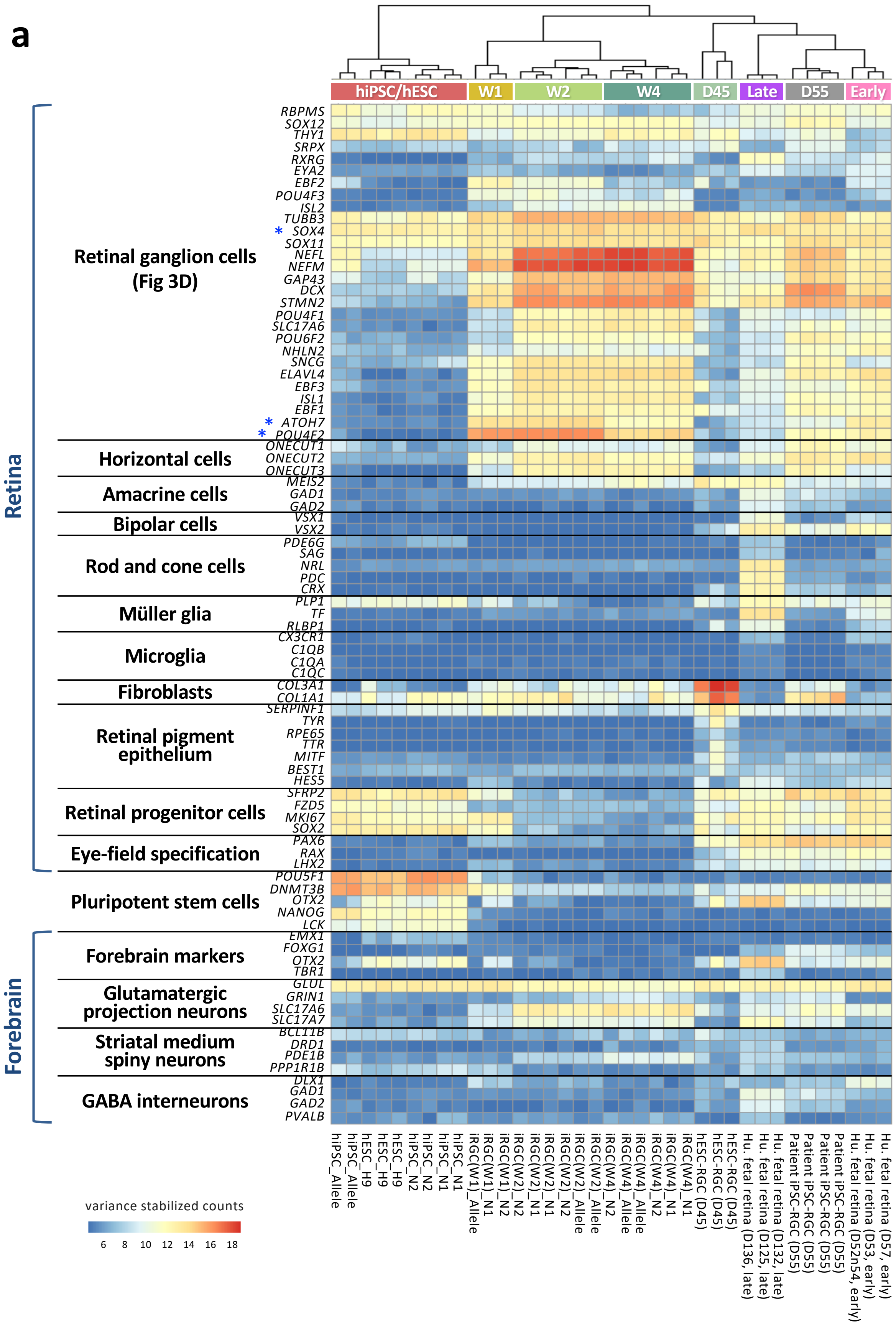

hiPSC/hESC
W1
W2
W4
D45
Late
D55
Early
Retina
Forebrain
Retinal ganglion cells (Fig 3D)
Horizontal cells
Amacrine cells
Bipolar cells
Rod and cone cells
Müller glia
Microglia
Fibroblasts
Retinal pigment epithelium
Retinal progenitor cells
Eye-field specification
Pluripotent stem cells
Forebrain markers
Glutamatergic projection neurons
Striatal medium spiny neurons
GABA interneurons
RBPMS
SOX12
THY1
SRPX
RXRG
EYA2
EBF2
POU4F3
ISL2
TUBB3
* SOX4
SOX11
NEFL
NEFM
GAP43
DCX
STMN2
POU4F1
SLC17A6
POU6F2
NHLN2
SNCG
ELAVL4
EBF3
ISL1
EBF1
* ATOH7
* POU4F2
ONECUT1
ONECUT2
ONECUT3
MEIS2
GAD1
GAD2
VSX1
VSX2
PDE6G
SAG
NRL
PDC
CRX
PLP1
TF
RLBP1
CX3CR1
C1QB
C1QA
C1QC
COL3A1
COL1A1
SERPINF1
TYR
RPE65
TTR
MITF
BEST1
HES5
SFRP2
FZD5
MKI67
SOX2
PAX6
RAX
LHX2
POU5F1
DNMT3B
OTX2
NANOG
LCK
EMX1
FOXG1
OTX2
TBR1
GLUL
GRIN1
SLC17A6
SLC17A7
BCL11B
DRD1
PDE1B
PPP1R1B
DLX1
GAD1
GAD2
PVALB
hiPSC_Allele
hiPSC_Allele
hESC_H9
hESC_H9
hESC_H9
hiPSC_N2
hiPSC_N2
hiPSC_N1
hiPSC_N1
iRGC(W1)_Allele
iRGC(W1)_N1
iRGC(W1)_N2
iRGC(W2)_N2
iRGC(W2)_N1
iRGC(W2)_N1
iRGC(W2)_N2
iRGC(W2)_Allele
iRGC(W2)_Allele
iRGC(W4)_N2
iRGC(W4)_Allele
iRGC(W4)_Allele
iRGC(W4)_N2
iRGC(W4)_N1
iRGC(W4)_N1
hESC-RGC (D45)
hESC-RGC (D45)
hESC-RGC (D45)
Hu. fetal retina (D136, late)
Hu. fetal retina (D125, late)
Hu. fetal retina (D132, late)
Patient iPSC-RGC (D55)
Patient iPSC-RGC (D55)
Patient iPSC-RGC (D55)
Patient iPSC-RGC (D55)
Hu. fetal retina (D52n54, early)
Hu. fetal retina (D53, early)
Hu. fetal retina (D57, early)
variance stabilized counts
6
8
10
12
14
16
18

Suppl Fig. 5 (continued)

b

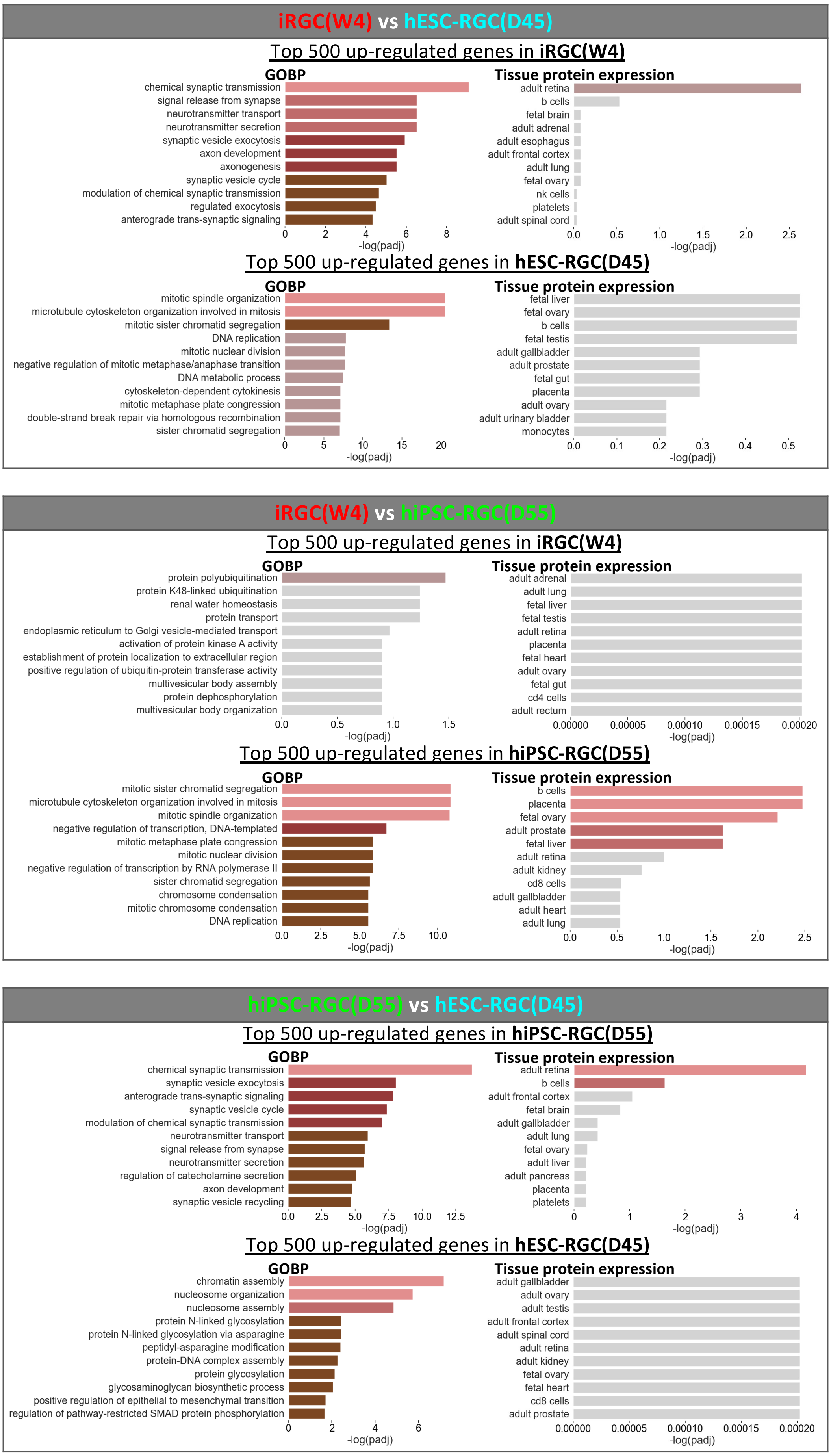

Fig S5. Marker gene expression and enrichment analysis in all samples. (**a**) A heatmap showing the expression of marker genes during retinal development and within retinal cell types in all samples. The Tet-on inducible genes are marked with asterisk (*). (**b**) A gene enrichment analysis was performed on the top 500 differentially upregulated genes (sorted by adjusted p-value and a fold change of ≥2). The eleven most significant groups are presented in terms of GOBP (left panel) and the tissue protein expression (right panel). Gray bars indicate no significant expression (padj > 0.05).

Suppl Fig. 6

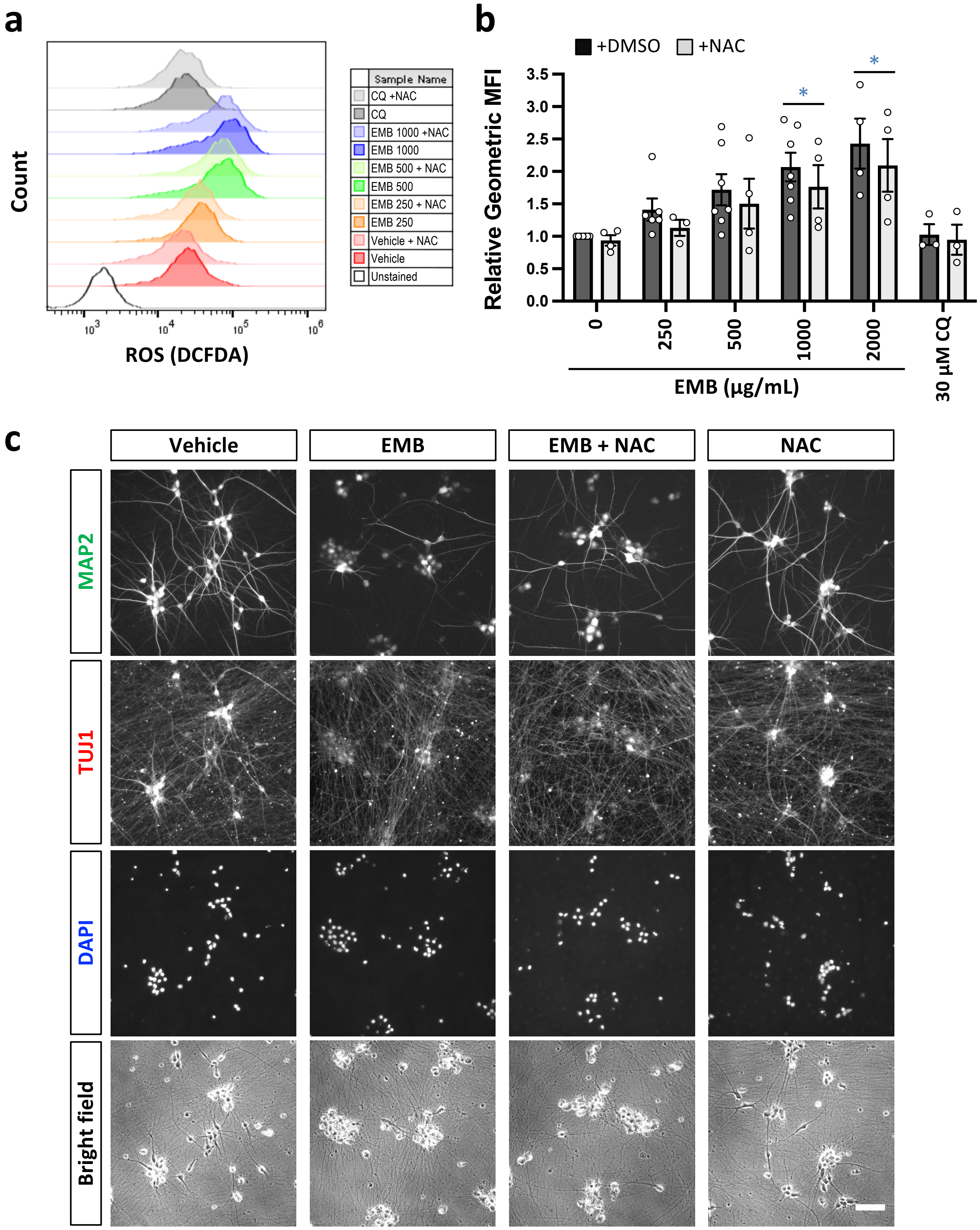
a
Count
ROS (DCFDA)
Sample Name
CQ +NAC
CQ
EMB 1000 +NAC
EMB 1000
EMB 500 + NAC
EMB 500
EMB 250 + NAC
EMB 250
Vehicle + NAC
Vehicle
Unstained
b
+DMSO
+NAC
Relative Geometric MFI
0
250
500
1000
2000
EMB (µg/mL)
30 µM CQ
c
Vehicle
EMB
EMB + NAC
NAC
MAP2
TUJ1
DAPI
Bright field

Fig S6. EMB-induced apoptosis and neurite degeneration in iRGC-ATOH7/BRN3B/SOX4 cells is able to be rescued by NAC. (**a**) iRGC cells were treated with various concentrations of EMB in the presence or absence of 500 μM NAC for 24 hrs. ROS generation was measured by flow cytometry using DCFDA staining. (**b**) Quantitative results of the geometric mean fluorescence intensity (MFI) of the treated cells relative to control non-treated cells. All results are presented as the mean ± SEM ($n = 4$ independent experiments; *: $p < 0.05$ by two-way ANOVA and Šídák's test). (**c**) The morphological changes in the iRGCs after 1000 μg/mL EMB treatment in the presence or absence of 500 μM NAC for 24 hrs and immunolabeled for MAP2 and TuJ1. Scale bar, 50 μm.

Table S1. Primers used for plasmid construction

Primer names	Sequence	Reference
EcoRI-Atoh7-Forward	5'-CCCCGAATTCATGAAGTCCTGCAAGCCCAGCG-3'	NM_145178
Atoh7-FLAG-XbaI-Reverse	5'-CCTCTCTAGACTTATCGTCGTCATCCTTGTAATCGGTGGCCATCTGGAAGGGCT-3'	
EcoRI-Brn2-Forward	5'-CCCCGAATTCATGGCGACCGCAGCGTCT-3'	NM_008899
Brn2-FLAG-XbaI-Reverse	5'-CCTCTCTAGACTTATCGTCGTCATCCTTGTAATCCTGGACGGGCGTCTGCACC-3'	
EcoRI-Brn3A-Forward	5'-CCCCGAATTCATGATGTCCATGAACAGCAAGCAG-3'	NM_006237
Brn3A-FLAG-XbaIReverse	5'-CCTCTCTAGACTTATCGTCGTCATCCTTGTAATCGTAAGTGGCAGAGAATTTCATCCGCT-3'	
EcoRI-Brn3B-Forward	5'-CCCCGAATTCATGATGATGATGTCCCTGAACAGCA-3'	NM_004575
Brn3B-FLAG-XbaI-Reverse	5'-CCTCTCTAGACTTATCGTCGTCATCCTTGTAATCAATGCCGGCGGAATATTTCATTCT-3'	
EcoRI-PAX6-Forward	5'-CCCCGGTCTCGAATTCATGCAGAACAGTCACAGCGGAGT-3'	NM_000280
PAX6-FLAG-XbaI-Reverse	5'-CCTCTCTAGACTTATCGTCGTCATCCTTGTAATCCTGTAATCTTGGCCAGTATTGAGACATATCAGGT-3'	
EcoRI-Irx6-Forward	5'-CCCCGAATTCATGTCCTTCCCACACTTTGGACAC-3'	NM_024335
Irx6-FLAG-XbaI-Reverse	5'-CCTCTCTAGACTTATCGTCGTCATCCTTGTAATCACCTGCTTCTGCTCCAGACGGG-3'	
EcoRI-SOX4-Forward	5'-CCCCGAATTCATGGTGCAGCAAACCAACAATGCCGAGAA-3'	NM_003107
SOX4-FLAG-XbaI-Reverse	5'-CCTCTCTAGACTTATCGTCGTCATCCTTGTAATCGTAGGTGAAAACCAGGTTGGAGATGCTGG-3'	
EcoR,Age-Ascl1-Forward	5'-GCAAGAATTCGAACCGGTGCCACCATGGAAAGCTCTGCCAAGATG-3'	NM_004316
Ascl1-FLAG-XbaIReverse	5'-GCAATCTAGACTTGTCATCGTCGTCCTTGTAATCGAACCAGTTGGTGAAGTCGAG-3'	
GSG-T2A-Forward	5'-CCCCTTTGGTCTCGAATTAACCGGTGGATCCGAATTCACATCTAGAGGAAGCGGAGAGGGCAGGGGAAGTCTTCTAACATGCGG-3'	
Zeo-PacI-Reverse	5'-TAGCTTAATTAATCAGTCCTGCTCCTCGGCCACGAAG-3'	
AgeI-Atoh7-Forward	5'-ATTCACCGGTATGAAGTCCTGCAAGCCCAGCG-3'	NM_145178
Atoh7-myc-Reverse	5'-AGAAGACTTCCCCTGCCCTCTCCGCTTCCCAGATCCTCTTCAGAGATGAGTTTCTGCTCGGTGGCCATCTGGAAGGGCT-3'	
myc-GSG-T2A-EcoRI-Reverse	5'-ACATGAATTCTGGGCCGGGATTTTCCTCCACGTCCCCGCATGTTAGAAGACTTCCCCTGCCCTCTCCGC-3'	
AgeI-Brn3B-Forward	5'-ATTCACCGGTATGATGATGATGTCCCTGAACAGCA-3'	NM_004575
Brn3B-myc-Reverse	5'-AGAAGACTTCCCCTGCCCTCTCCGCTTCCCAGATCCTCTTCAGAGATGAGTTTCTGCTCAATGCCGGCGGAATATTTCATTCT-3'	

Table S2. The antibodies and dilutions used in this study

Antibody Names	Manufacturer	Cat. #	Dilution
Mouse anti-FLAG M2 monoclonal antibody	Sigma-Aldrich	F3165	1:5000
Mouse anti-c-myc monoclonal antibody	Developmental Studies Hybridoma Bank (DSHB)	AB_2266850	1:5000
Mouse anti-β-tubulin monoclonal antibody	Developmental Studies Hybridoma Bank (DSHB)	AB_2315513	1:5000
Mouse anti-glyceraldehyde-3-phosphate dehydrogenase monoclonal antibody	Millipore	MAB374	1:5000
Mouse anti-neuronal class III β-tubulin monoclonal antibody	Covance	MMS-435P	1:1000
Rabbit anti-microtubule-associated protein 2 antibody	Millipore	AB5622	1:2000
Rabbit anti-Islet 1 polyclonal antibody	abcam	ab20670	1:2000
Mouse anti-Brn3a polyclonal antibody	Millipore	AB5945	1:500
Mouse anti-Tau antibody (clone Tau-5)	Sigma-Aldrich	MAB361	1:2000
Rabbit anti-microtubule-associated protein 1 light chain 3 α polyclonal antibody	Proteintech	12135-1-AP	1:3000
Rabbit anti-p62/SQSTM1 polyclonal antibody	Proteintech	18420-1-AP	1:5000
Rabbit anti-caspase-3 antibody	Cell Signaling Technology	#9662S	1:2000
Rabbit anti-cleaved caspase-3 (Asp175) (5A1E)	Cell Signaling Technology	#9664S	1:2000
Goat anti-mouse IgG-HRP	Bethyl laboratories	A90-216P	1:5000
Goat anti-rabbit IgG-HRP	Bethyl laboratories	A120-201P	1:5000
Rhodamine (TRITC)-conjugated goat anti-mouse IgG (H+L)	Jackson ImmunoResearch	115-025-146	1:1000
Goat anti-mouse IgG, DyLight™ 488 conjugated highly cross-absorbed	Thermo Scientific	35503	1:500
Goat anti-rabbit IgG H&L DyLight® 550	abcam	ab96900	1:500

Table S3. Primers and TaqMan probes for qPCR analysis

Gene	Sequence	Probe	Reference
ATOH7 (3' UTR)	(F) 5'-CAGACCTATGGACGCAATCA-3'<br>(R) 5'-CAACCCATTCACAAGATCCAT -3'	#63	NM_145178
RBPMS	(F) 5'-TGTGCCCTTAGAAAGCCCTA-3'<br>(R) 5'-CACACTTGGCACACAGTTGA-3'	#78	NM_001008710.3
POU4F1 (BRN3A)	(F) 5'-CTCCCTGAGCACAAGTACCC-3'<br>(R) 5'-CTGGCGAAGAGGTTGCTC -3'	#78	NM_006237.4
POU4F2 (BRN3B) (5' UTR)	(F) 5'-CCGAGATCAGGCGTACAGAG-3'<br>(R) 5'-GGGCTGTGCGAAGTTGAG -3'	#70	NM_004575.3
ISL1	(F) 5'-AAGGACAAGAAGCGAAGCAT-3'<br>(R) 5'-TTCCTGTCATCCCCTGGATA -3'	#66	NM_002202.2
SOX4 (5' UTR)	(F) 5'-CCAACTCCTTAGTGCCGATT-3'<br>(R) 5'-CCTCTCCTACAATGCAAAGCA-3'	#1	NM_003107.3
EBF1	(F) 5'-AGCTGCCAACTCCCCCTAT-3'<br>(R) 5'-GGGAGGCTTGTGGAGGAG -3'	#42	NM_001290360.2
RBFOX3 (NEUN)	(F) 5'-CCCTCCGACCCTACAGAGA-3'<br>(R) 5'-CCACGTCTAAAATTTTTCCGAAT -3'	#66	NM_001082575.1
TUBB3 (TUJ1)	(F) 5'-GCAACTACGTGGGCGACT-3'<br>(R) 5'-ATGGCTCGAGGCACGTACT-3'	#78	NM_006086.3
POU5F1 (OCT4)	(F) 5'-CTTCGCAAGCCCTCATTTC-3'<br>(R) 5'-GAGAAGGCGAAATCCGAAG -3'	#60	NM_002701.4
SLC17A7 (VGLUT1)	(F) 5'-AGAGCGCGAAACTCATGAAC-3'<br>(R) 5'-GCAGCAGGTAGAACGTCCAG -3'	#29	NM_020309.4
SLC17A6 (VGLUT2)	(F) 5'-AATCACTCGGCCAGATCTACA-3'<br>(R) 5'-CGTCAGCTCGATTGTCTCC-3'	#67	NM_020346.3
TH	(F) 5'-TCAGTGACGCCAAGGACA-3'<br>(R) 5'-GTACGGGTCGAACTTCACG-3'	#42	NM_199292.3
GAD1	(F) 5'-CAACTTGGAGCTCTCTGACCA-3'<br>(R) 5'-TGAAAAATCGAGGATGACCTG -3'	#1	NM_013445.3
CHAT	(F) 5'-GGAGCCTGAGCACGTCAT-3'<br>(R) 5'-CGGAAATTAATGACAACATCCA -3'	#40	NM_020984.4
TPH1	(F) 5'-GCTTCTCTTGGCGCTTCA-3'<br>(R) 5'-ACCAAACTCCACAGTGAAAAAGT-3'	#75	NM_004179.3
RPL13A	(F) 5'-CAAGCGGATGAACACCAAC-3'<br>(R) 5'-TGTGGGGCAGCATACCTC-3'	#28	NM_012423.4
